# Characterization of the Rapid Proteolytic Shedding of Murine L-Selectin

**DOI:** 10.1155/2001/91831

**Published:** 2001

**Authors:** Li-Chao Zhao, John B. Edgar, Morris O. Dailey

**Affiliations:** The Interdisciplinary Program in Immunology and the Departments of Pathology and Microbiology The University of Iowa College of Medicine Iowa City Iowa 52242 USA

**Keywords:** intercellular adhesion, L-selectin, metalloprotease, shedding

## Abstract

The structural requirements for L-selectin shedding were studied in murine leukocytes. Upon activation, L-selectin on both lymphocytes and neutrophils undergoes cleavage by a membrane metalloprotease, resulting in the generation of a soluble ectodomain and a membrane- retained 6 kD fragment. Radiochemical sequencing demonstrated a cleavage site in the membrane-proximal region (MPR) between R321 and S322, which is homologous to the human site. Although intact neutrophil L-selectin is larger, it is cleaved at the same, or very close, site. Analysis of several transfectants expressing L-selectin point mutations and chimeric constructs suggest that, like human shedding, the proteolytic process has relatively loose sequence specificity for the substrate site. In addition, some constructs are susceptible to slow constitutive cleavage, but their shedding does not increase upon PMA stimulation, showing that basal and activated shedding are separable processes. Insertion of the 15 amino acid MPR into murine B7.2 conferred upon this molecule susceptibility to constitutive shedding. PMA stimulation results in little or no acceleration of down regulation of this molecule. These results suggest that recognition of both the membrane-proximal cleavage site and of a site distant from the MPR are required for maximal induction of L-selectin shedding.


*Developmental Immunology*, 2001, Vol. 8(3-4), pp. 267-277
Reprints available directly from the publisher
Photocopying permitted by license only

© 2001 OPA (Overseas Publishers Association)
N.V. Published by license under
the Harwood Academic Publishers imprint,
part of The Gordon and Breach Publishing Group,
a member of the Taylor and Francis Group

### Characterization of the Rapid Proteolytic Shedding of Murine L-Selectin

LI-CHAO ZHAO, JOHN B. EDGAR and MORRIS O. DAILEY*

*The Interdisciplinary Program in Immunology and the Departments of Pathology and Microbiology, The University of Iowa College of Medicine Iowa City, Iowa 52242, U.S.A*

The structural requirements for L-selectin shedding were studied in murine leukocytes. Upon activation, L-selectin on both lymphocytes and neutrophils undergoes cleavage by a membrane metalloprotease, resulting in the generation of a soluble ectodomain and a membrane-retained 6 kD fragment. Radiochemical sequencing demonstrated a cleavage site in the membrane-proximal region (MPR) between R321 and S322, which is homologous to the human site. Although intact neutrophil L-selectin is larger, it is cleaved at the same, or very close, site. Analysis of several transfectants expressing L-selectin point mutations and chimeric constructs suggest that, like human shedding, the proteolytic process has relatively loose sequence specificity for the substrate site. In addition, some constructs are susceptible to slow constitutive cleavage, but their shedding does not increase upon PMA stimulation, showing that basal and activated shedding are separable processes. Insertion of the 15 amino acid MPR into murine B7.2 conferred upon this molecule susceptibility to constitutive shedding. PMA stimulation results in little or no acceleration of down regulation of this molecule. These results suggest that recognition of both the membrane-proximal cleavage site and of a site distant from the MPR are required for maximal induction of L-selectin shedding.

*Keywords:* intercellular adhesion, L-selectin, metalloprotease, shedding

*Abbreviations:* LN, lymph node, HEV, high endothelial venule, MPR, membrane-proximal region, MRF, membrane-retained fragment

### INTRODUCTION

The adhesion molecule L-selectin expressed on the surface of B and T lymphocytes mediates the initial interaction of lymphocytes with high endothelial venules (HEV) in the T-dependent zone of lymph nodes (LN). Lymphocytes require L-selectin to home to LN and this molecule therefore is a central regulator of lymphocyte recirculation in vivo. Thus, lymphocytes from genetically L-selectin-deficient mice are completely unable to home from the bloodstream into LN, the LN are systemically atrophic, and immune responses to peripherally located antigens are therefore suppressed or delayed (Arbones *et al.*, 1994; Catalina *et al.*, 1996; Steeber *et al.*, 1996; Tedder *et al.*, 1995; Xu *et al.*, 1996). The expression of L-selectin is regulated at a series of steps, including modulation of transcription, mRNA stability, and post-translationally at the cell surface (Chao *et al.*, 1997). Post-translational regulation of L-selectin is

* Corresponding author. Tel. 319–335–8184; FAX 319–335–6555

much more rapid than regulation at the other steps and involves rapid proteolytic cleavage and shedding of the membrane molecule (Huang *et al.*, 1990; Jung and Dailey, 1990). A similar mechanism causes L-selectin down regulation in neutrophils (Jutila *et al.*, 1990; Kishimoto *et al.*, 1989). Lymphocyte L-selectin shedding is induced upon treatment with PMA (Jung and Dailey, 1990), calmodulin antagonists (Kahn *et al.*, 1998), or after cross-linking CD3 with antibody (Chao *et al.*, 1997), suggesting possible roles of PKC and calcium-calmodulin-dependent kinases in regulating shedding. While the precise function of L-selectin shedding is as yet unclear, it may have a role in regulating transendothelial migration of lymphocytes and some evidence shows that it may affect leukocyte rolling on endothelium (Hafezi-Moghadam and Ley, 1999; Walcheck *et al.*, 1996).

The protease responsible for L-selectin shedding has been shown to be a novel integral membrane $Zn^{++}$-dependent metalloprotease on the surface of the cell (Black *et al.*, 1997; Moss *et al.*, 1997). Upon activation, this enzyme recognizes L-selectin and then cleaves it just external to the plasma membrane. The same enzyme appears to be responsible for the shedding of several other membrane proteins, from a variety of cell types, including TNF-α, p75TNFR, proTGFα, and β-amyloid precursor protein (Buxbaum *et al.*, 1998; Peschon *et al.*, 1998). One curious feature of this process is that it has very loose substrate specificity, such that variations in amino acid sequence in and adjacent to the cleavage sites do not effectively block proteolysis (Hooper *et al.*, 1997). Thus, most mutations in the membrane-proximal region (MPR) do not prevent the shedding of human L-selectin (Chen *et al.*, 1995; Migaki *et al.*, 1995). This enzymatic process, however, cannot be completely promiscuous because most cell surface proteins are *not* cleaved. It is therefore unclear how this L-selectin sheddase recognizes L-selectin or the other proteins upon which it acts.

In the experiments reported herein, we examined L-selectin shedding from normal mouse lymphocytes, determined the cleavage site, and compared it to cleavage in neutrophils. Several mutant L-selectin transfectants were then used to examine the specificity of the murine cleavage process and the mechanism by which this cleavage is recognized. The data show that murine L-selectin is cleaved at a site homologous to its human counterpart. In addition, the slow constitutive shedding and rapid shedding in activated cells are separable processes. While presence of the short MPR sequence is sufficient for the basal level of cleavage, it is not sufficient to confer upon a heterologous protein maximal susceptibility to PMA-induced shedding. This implies that a region of L-selectin distant from the MPR is recognized by the protease, or a protease-associated molecule, and that this recognition is important in the normal enzymatic cleavage of L-selectin in PMA-stimulated cells.

### MATERIALS AND METHODS

### Cell Culture and Antibodies

An L-selectin-negative clone of the mouse T lymphoma cell line, TK-1, was maintained in RPMI 1640 supplemented with 10% FCS, penicillin (1,000 U/ml), streptomycin (1,000 U/ml), glutamine (20 mM), HEPES (10 mM) and 2-mercaptoethanol (50 μM). The stable transfectant lines were maintained in the complete medium containing 0.5 mg/ml G418 (Calbiochem, La Jolla, CA). The following antibodies were used in this study: MEL-14: rat IgG2a anti-mouse L-selectin; T28: mouse IgG2 anti-mouse L-selectin of $Ly22^+$ allotype; GL-1: rat IgG2a anti-mouse B7.2. These mAbs were prepared from tissue culture supernatant and used conjugated to biotin or phycoerythrin (PE) using established methods (Mobley and Dailey, 1992). PE-labeled streptavidin and PE-conjugated donkey anti-rat Ig antibody were purchased from Southern Biotechnology Associates, Inc. (Birmingham, AL).

### Neutrophil Preparation

Bone marrow cells from Balb/c mice were underlayed with 50% Percoll (Sigma, St. Louis, MO) and then centrifuged at 2,600 rpm for 30 min. Cell pellets were washed and resuspended in HBSS. Then, cells were further separated on 63% Percoll as above. The cell pellet was washed and the purity of neutrophils was evaluated by Wright staining.

### cDNA Constructs and Site-Specific Mutagenesis

Site-specific mutations and chimeric molecules were generated by the sequential PCR method (Saiki *et al.*, 1988). Briefly, mutations were introduced using two inner complementary primers coding for the new amino acids. For the first PCR step, individual outer primers that are complementary to either the upstream sequence or downstream sequence were used with individual inner primers to amplify a DNA fragment from the template. Then the two amplified fragments were annealed and used as a template for the second PCR reaction, in which only the two outer primers were used. The final mutated PCR products were digested with appropriate enzymes and used to replace the equivalent DNA fragment in the original wt cDNA construct.

L-selectin cDNA in the Rsv.Sneo vector, into which mutated fragments were inserted, had the wt sequence of the Ly22$^-$ allotype. The DNA fragment from Apa I to BamH I was amplified in the sequential PCR reactions to generate L-selectin mutants. Wild type L-selectin and all L-selectin mutant cDNAs with the Ly22$^-$ allotype were removed with Sal I and BamH I digestion and ligated into the pHSE vector (Pircher *et al.*, 1989; kindly provided by Dr. D. Raulet) digested with the same enzymes. The sequence of all L-selectin mutants was verified by DNA sequencing.

A chimeric molecule with the MPR of L-selectin inserted between the extracellular and transmembrane domains of the mouse B7.2 molecule were also generated using the sequential PCR method as follows. First, the sequence encoding the FLAG octapeptide (N-DYKDDDDK-C) was added to the carboxy terminus of B7.2 by amplifying the EcoR V to Xba I fragment of B7.2 cDNA in pCDNA3 vector with a downstream primer containing this FLAG sequence in a single step PCR reaction. Then this flag-tagged B7.2 cDNA was used as a template to generate the chimeric cDNA. A DNA fragment encoding the MPR of L-selectin was inserted between the transmembrane domain and extracellular domain of B7.2 by amplifying the EcoR V to Xba I fragment of flag-tagged B7.2 cDNA to generate the B7.2/MPR chimeric cDNA. B7.2 and B7.2/MPR cDNAs were removed from pCDNA3 by Apa I and Kpn I digestion and blunt-ended. They were then ligated into the pHSE vector digested with Sma I and EcoR V.

### Stable Transfection

DNA fragments between the BssH II sites, which contains the cDNA encoding Ly22$^-$ wt L-selectin, L-selectin mutants and the chimeric molecules, were removed from the pHSE constructs and purified by gel extraction. The pCDNA3 vector containing the G418 resistance marker was linearized by Sca I digestion. 10 μg of the DNA fragments containing these cDNAs were mixed with linearized pCDNA3 vector at a molar ratio 5:1 and were transferred to 0.5 ml L-selectin-negative TK-1 cell suspension at $1 \times 10^7$ cells/ml in complete medium. The cell suspension with the DNA constructs in a 0.4 cm electrode gap cuvette was electroporated at 250 V, 960 μF at room temperature using a Gene Pulser (Bio-Rad Laboratories, CA). The cells were resuspended and grown in complete medium for two days. They were then cloned and selected at 5000 cells/well in 96-well plates in complete medium containing 2 mg/ml G418. Ten to 20 clones from each transfection were tested by flow cytometry analysis with PE-conjugated MEL-14 or GL-1 followed by PE-conjugated goat anti-rat Ig antibodies for L-selectin or B7.2 expression, respectively. The clones positive for L-selectin mutants were also tested for their Ly22 allotype, to ensure that endogenous wt L-selectin genes were not expressed, by staining the cells with biotin-conjugated T28 antibodies followed by PE-conjugated avidin. Three clones positive for MEL-14 or GL-1, but negative for T28, were used in the following studies.

### Flow Cytometric Analysis of Cell Surface Protein Shedding

Cells (5x $10^5$) were resuspended in 0.5 ml Hank's balanced salt solution (HBSS) containing 10% horse serum (FACS buffer) in test tubes. For testing PMA-induced shedding, 100 ng/ml PMA was added to the tubes and the cell suspension was incubated at 37°C for the time indicated. For testing the effect of the metalloprotease inhibitor (RO31–9790, kindly provided by Dr. Ann Ager, London) on PMA-induced shedding of L-selectin mutants, 50 μM RO31–9790 was added to the tubes and the cell suspension was pre-incubated at room temperature for 20 min. Then, 100 ng/ml PMA was added and the cell suspension was further incubated at 37°C for 30 min. For all experiments, tubes with the same concentration of DMSO added as the experimental tubes were used as controls. Then the cells were wash once and stained with PE-conjugated MEL-14 antibody for L-selectin, or with GL-1 followed by PE-coupled goat anti-rat Ig antibody for B7.2. PE-conjugated normal rat Ig and isotype-matched antibody were used as negative controls. The stained cells were analyzed on a FACS Vantage (Becton-Dickinson, Mountain View, CA).

### Preparation of Anti-Peptide Antibodies

A 17-mer oligopeptide corresponding to the cytoplasmic domain of mouse L-selectin (NH2-RRLKKG-KKSQERMDDPY-COOH) conjugated to diphtheria toxoid (Chiron Mimetopes, Australia) was used to immunize rabbits. Antibodies were purified by passing the resulting antisera over a column containing the cytoplasmic domain peptide.

### Western Blot Analysis of L-selectin Shedding

Normal lymphocytes, neutrophils, or the transfected TK-1 cells were left unstimulated or stimulated with 100 ng/ml PMA at 37°C for the times indicated. Depending on the expression level of L-selectin, 1 × $10^7$ normal lymphocytes or 2 to 4 × $10^7$ transfectants were lysed in 1 ml lysis buffer (2% Triton X-100, PBS, 0.1% azide and 5 mg/ml aprotinin, 2.5 mg/ml pepstatin, 5 mg/ml leupeptin, 10 mM iodoacetamide, 2 mM PMSF, 2 mM EDTA as protease inhibitors). Nuclei and cell debris were removed by centrifugation at 20,800xg for 30 min. at 4°C. Then 100 μl 10% sodium deoxycholate and 10 μl 10% SDS were added to the clarified lysates at final concentrations of 1% and 0.1%, respectively. The lysates were precleared twice with 20 μl non-immune rabbit IgG-sepharose beads (3 mg antibody/ml beads), followed by immunoprecipitation with 10 μl sepharose beads coupled with the anti-L-selectin cytoplasmic domain peptide antibody (1 mg antibody/ml beads) for 2 h at 4°C. Immunocomplexes were washed twice in the lysis buffer and twice in PBS containing 0.1% Triton X-100. The bound antigen was eluted in 25 or 45 μl of low-pH elution buffer (100 mM glycine pH 2.5, 0.15 M NaCl, 0.1% Triton X-100) for 1 h at 4°C. Then 20 or 40 μl of eluant was collected and neutralized with 2 or 4 μl of neutralization buffer (2 M Tris-Cl pH7.4). 15 μl of the eluant was loaded on 10%-20% Tricine-SDS gels (Novex, San Diego, CA) and separated under reducing conditions. Proteins separated on the gel were electrotransferred to a 9 cm x 11 cm Protran nitrocellulose membrane (Schleicher and Schuell, Keene, NH) at 25 V overnight. The membrane was blocked with 5% non-fat dry milk for 1 h, then was washed with TTBS (50 mM Tris-HCl pH 7.4, 0.15 M NaCl, 0.1% Tween-20, 0.1% azide). After washing, the membrane was incubated with 5 μg/ml anti-L-selectin cytoplasmic domain peptide antibody in 40 ml blotting buffer (TTBS containing 1% Gelatin) for 2 h, followed by 1:40,000 horseradish peroxidase-coupled donkey anti-rabbit Ig conjugate (Jackson ImmunoResearch Laboratories, West Grove, PA) in 40 ml blotting buffer. The membrane was washed twice with TTBS for 10 min after each step. The membranes were visualized by LumiGLO Chemiluminescent Substrate (Kirkegaard and Perry Laboratories, Gaithersburg, Maryland) and exposure to film.

### Radiochemical Sequence Analysis

Radiolabeled membrane-retained L-selectin cleavage fragment was purified on a 2D gel and then sequenced, as described below. 1.3 × $10^9$ Balb/c spleen cells treated with 100 ng/ml PMA at 37°C for 15 min were resuspended at 1 × $10^8$ cells/ml in the lysis buffer used in the western blot analysis. Cell lysate was precleared twice with 150 μl non-immune rabbit IgG-Sepharose beads and then immunoprecipitated with 80 μl of Sepharose beads coupled with the anti-L-selectin cytoplasmic domain peptide antibody. The beads were washed three times with PBS containing 2% Triton X-100 and 0.5% SDS, then once with PBS containing 0.1% Triton X-100. Bound antigen was eluted with 450 μl low-pH elution buffer. The eluted protein was labeled with 1.5 mCi $^{125}$I (Amersham Corp., Arlington Heights, IL) using IODO-GEN (Pierce, Rockford, IL) as per the manufacturer's recommendations. After labeling, proteins were precipitated and washed twice with cold 10% trichloroacetic acid, washed twice with acetone, and the dried material resuspended in isoelectric-focusing (IEF) gel sample buffer. Proteins were first separated on an IEF tube gel (pH 3 to 10) and then separated in the second dimension on a 10%-20% Tricine-SDS PAGE gel (Integrated Separation Systems, Natic, MA). Proteins on this gel were transferred to a Westran PVDF membrane (Schleicher and Schuell, Keene, NH). Air-dried membrane was exposed to Kodak XAR5 film. After the film was developed, it was aligned with the membrane and the area corresponding to the radioactive spot of the 6 kD membrane-retained fragment (MRF) shown on the film was excised from the membrane. The labeled MRF was sequenced by automated Edmann degradation in the University of Iowa Protein Core Laboratory. In preliminary experiments, the identity of the $^{125}$I-labeled L-selectin MRF was confirmed by western blotting the radiolabeled material as described above (data not shown).

### RESULTS

Treatment of lymphocytes with phorbol esters that activate PKC causes L-selectin shedding from the cell surface. Figure 1a shows the decrease in selectin expression that occurs when mouse lymph node cells are treated with PMA, with 96% loss in 30 minutes. Pre-treatment of the cells with RO-319790, a metalloprotease inhibitor (Preece *et al.*, 1996), blocks this down regulation, confirming that this is mediated by the selectin sheddase. PMA-treated and untreated cells were immunoprecipitated with polyclonal antibody specific for the cytoplasmic domain of L-selectin and then examined by western blot analysis, blotting the membrane with the same antibody (Figure 1b). In untreated cells, there is a 95 kD band corresponding to intact L-selectin. In addition, there is a 6 kD band which represents the protein fragment that remains in the membrane after release of the soluble ectodomain. This consists of the cytoplasmic and transmembrane domains and that portion of the ectodomain that is proximal to the cleavage site. After PMA treatment, the gp95 band decreases in intensity, as expected, while that of the membrane-retained fragment increases, reflecting its generation during proteolytic cleavage.

PMA also induces the rapid down regulation of L-selectin in murine neutrophils, as shown in Figure 2a. The intact molecule expressed on neutrophils has a higher molecular weight than that on lymphocytes, reflecting different glycosylation (Figure 2b; see also Kishimoto *et al.*, 1989). Cleavage of this molecule results in generation of the same size MRF as with lymphocytes, demonstrating that they are cleaved at the same, or very close, sites on the intact molecule.

The human L-selectin cleavage site has been determined in transfectant cell lines. We performed experiments to determine the precise cleavage site of the mouse protein in normal lymphoid cells. We were unable to internally label normal spleen cells with radioactive amino acids to a sufficient specific activity to perform protein sequencing, so the following approach was used. Normal spleen cells were treated with PMA to induce shedding and membrane lysates were immunoprecipitated with anti-cytoplasmic peptide antibody. Then, the resulting immunoprecipitated protein was labeled with $^{125}$I and separated on a 2D gel (Figure 3a). This resulted in a highly purified sample of radiolabeled membrane-retained 6kD fragment, which was then subjected to Edmann degrada-

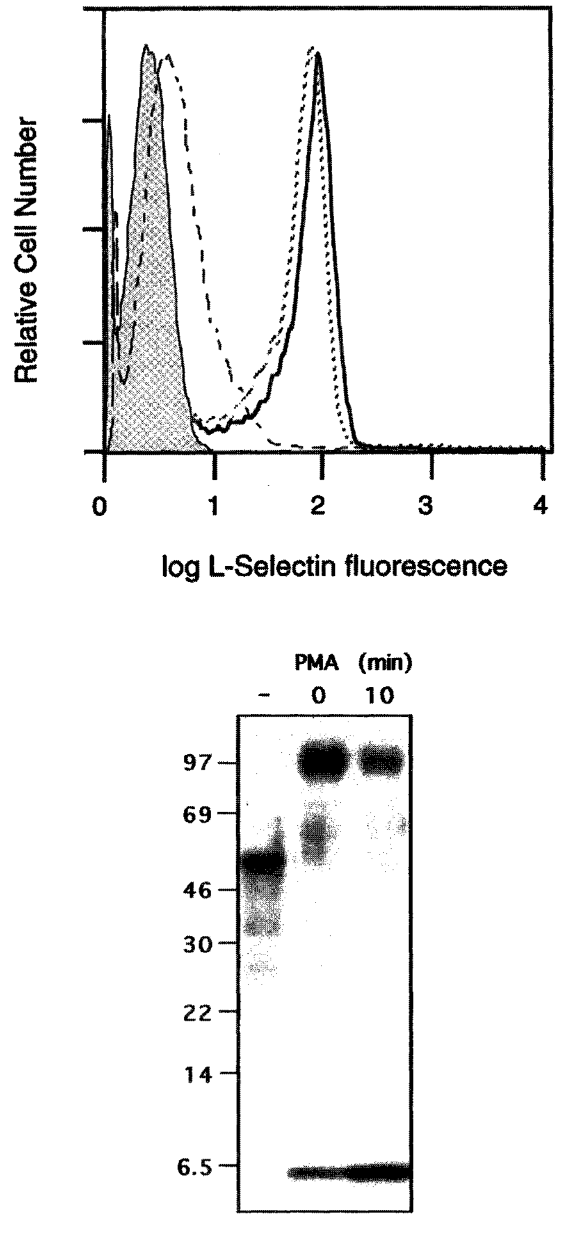

FIGURE 1 Shedding of L-selectin on normal lymphocytes. (a) FACS analysis of L-selectin expression on lymph node lymphocytes; untreated (solid line), PMA-treated (dashed line), or PMA-stimulated preceded by pre-treatment with the protease inhibitor RO31–9790 (dotted line). Negative control staining with normal rat Ig is shown in the shaded histogram. (b) Western blot analysis of lymphocytes immunoprecipitated and detected with the anti-cytoplasmic domain antibody. Lane 1, non-immune rabbit Ig negative control; lane 2, untreated lymphocytes; lane 3, PMA-treated for 10 minutes prior to lysis

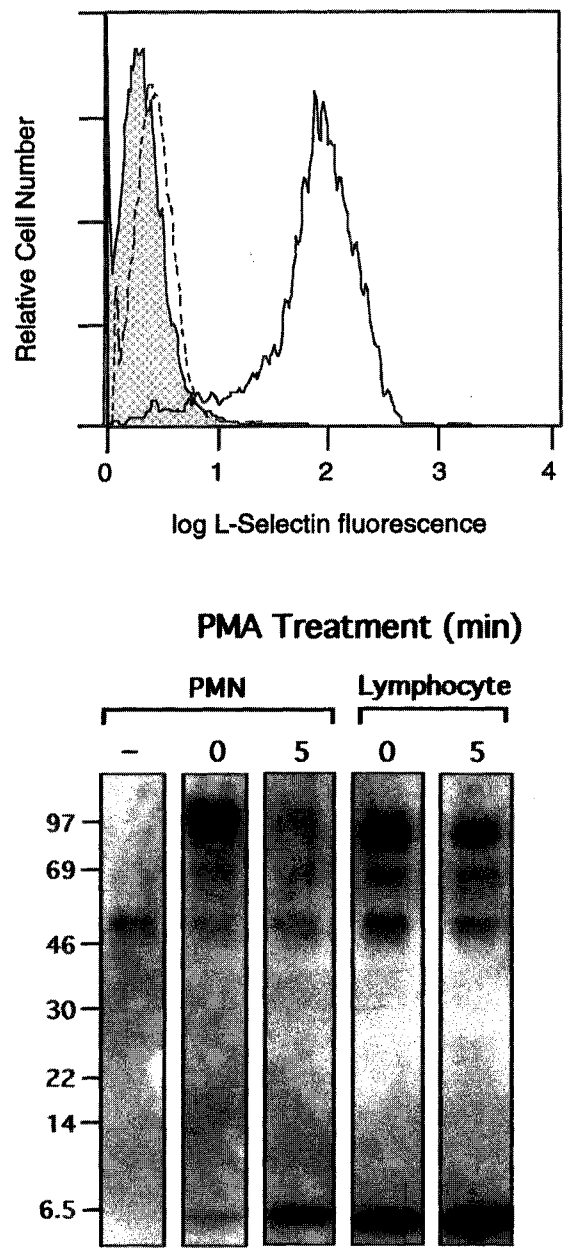

FIGURE 2 Shedding of L-selectin on neutrophils. (a) Purified neutrophils from bone marrow were left untreated (solid line) or treated with PMA for 15 min. (dashed line). Shaded histogram, negative control staining with PE-coupled normal rat Ig. (b) Western blot analysis of L-selectin shedding on neutrophils. Neutrophils or normal spleen lymphocytes were left untreated or treated with PMA for 5 min, as indicated. Cells were lysed and analyzed as described in Materials and Methods

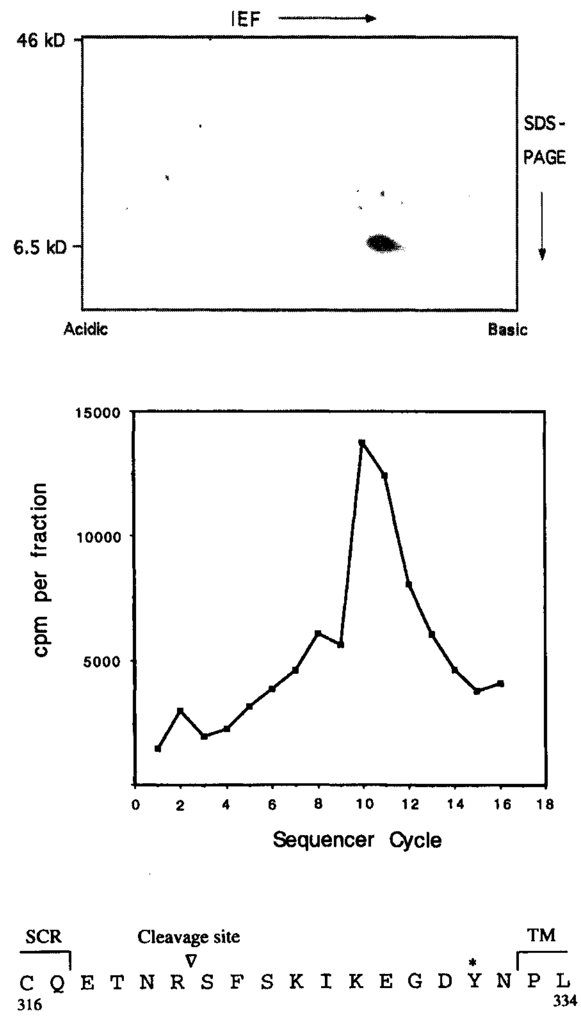

FIGURE 3 Determination of the cleavage site of murine L-selectin by radiochemical sequencing. (a) 2-D gel analysis of the immunoprecipitated, $^{125}$I-labeled membrane-retained fragment of L-selectin used for sequencing. (b) Results of MRF sequencing. The plot shows the $^{125}$I radioactivity of each fraction obtained from the automated sequencer. (c) Diagram of the cleavage site as determined by the position of the radioactive peak corresponding to the labeled tyrosine residue in the MRF. The position of the labeled tyrosine is marked by$^{*}$. The end of the second SCR domain and beginning of the transmembrane domain are indicated tion in a protein sequencer. The $^{125}$I radioactivity was then measured in each fraction. As shown in Figure 3b, there is a peak of radioactivity at position 10. Comparing this to the known sequence of L-selectin, and counting back from the labeled tyrosine, these data show that the cleavage site is between R321 and S322 (Figure 3c). This is the same site as the human homologue, which cleaves between K283 and S284 (Kahn *et al.*, 1994).

In order to examine how the protease recognizes its substrate, we examined the cleavage of several site-specific mutants and chimeric molecules. Using the sequence information above, mutations in the amino acids adjacent to the cleavage site were constructed and expressed in the murine cell line TK-1. As shown in Figure 4, wildtype L-selectin transfectants shed in response to PMA, and this shedding is inhibited by RO-319790. The mutants with amino acid substitutions of R321I and S322I are also shed after PMA treatment, in keeping with the loose substrate specificity shown for the human molecule (Chen *et al.*, 1995; Migaki *et al.*, 1995). In contrast to these mutations, replacement of the phenylalanine with a proline (F323P) dramatically inhibits PMA-induced down regulation; even after 30 minutes, no decrease in selectin expression is seen (Figure 4).

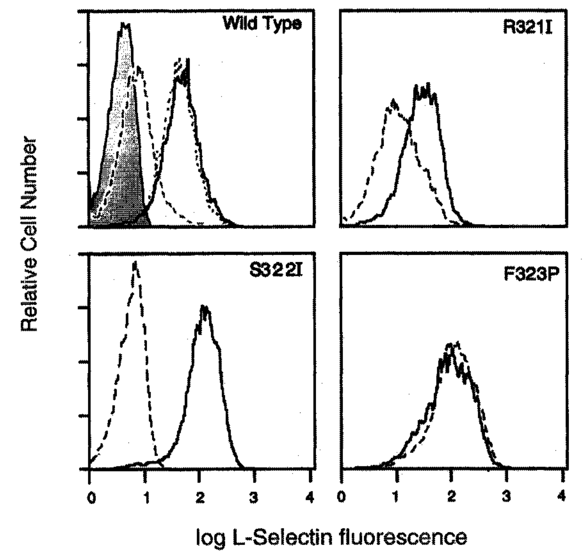

FIGURE 4 FACS analysis of PMA-induced shedding of point mutants of L-selectin. TK-1 cells transfected with cDNA of wt L-selectin, R321I, S322I, F323P were treated with PMA, with or without protease inhibitor, and then analyzed as described in Fig. 1a

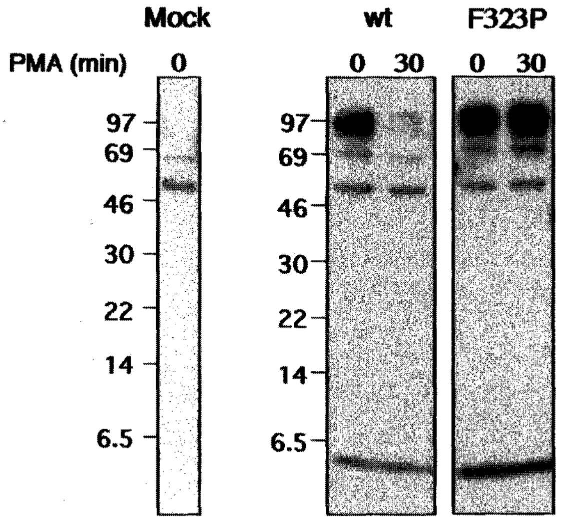

FIGURE 5 Western blot analysis of preteolytic cleavage of the proline substitution mutant. Untreated or PMA-treated F323P mutant transfectant was immunoprecipitated and probed as described in Materials and Methods. Similar analysis performed on wildtype and mock-transfected cells are shown for comparison

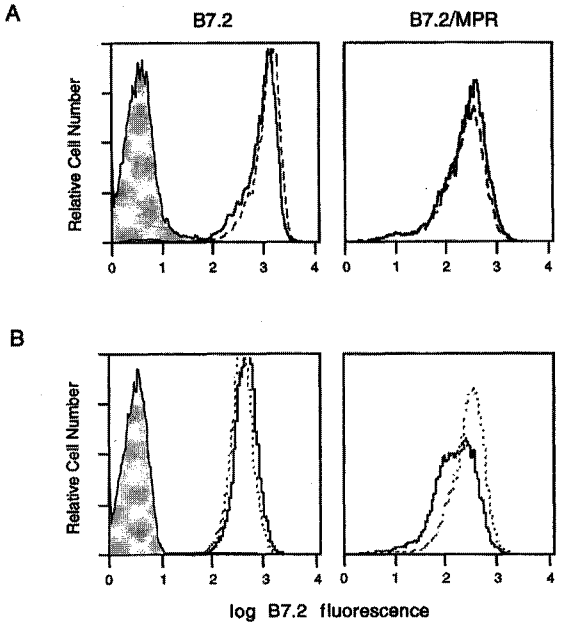

FIGURE 6 Constitutive shedding of B7.2 containing the MPR of L-selectin. (a) TK-1 cells transfected with wt B7.2 and B7.2/MPR chimeric cDNA (as indicated) were treated with PMA and stained with anti-B7.2. (b) B7.2 and B7.2/MPR transfectants were incubated in RO31–9790 (dotted line) for 2h, followed by flow cytometric analysis with anti-B7.2. The solid line shows the fluorescence of cells treated with the inhibitor solvent, DMSO, only

Western blot analysis shows that the wildtype L-selectin transfectant expresses the normal gp95 band, which down regulates upon stimulation, and the same 6 kD MRF as seen in normal lymphocytes (Figure 5). Surprising results, however, were obtained with the F323P mutant. Western blots of F323P show a dark gp95 band, as expected, but also show considerable amount of the 6 kD MRF. Treatment with PMA neither induces a decrease in gp95 or any change in the MRF. Thus, while shedding of this molecule does not increase with PMA stimulation, it is subject to an invariant basal level of proteolytic cleavage.

The simplest structural unit that might be recognized by the protease is, of course, the region around the cleavage site itself, although that recognition would have to be relatively relaxed. In order to determine whether the MPR by itself is sufficient for recognition, a chimeric cDNA was constructed in which the MPR was inserted into the membrane-proximal sequence of murine B7.2. Transfectants expressing the B7.2/MPR chimera or wildtype B7.2 were treated with PMA and the degree of down regulation was determined by immunofluorescence analysis with anti-B7.2 antibody. As shown in Figure 6a, both constructs are expressed well. B7.2 is not down regulated in response to PMA treatment, as expected. In multiple experiments, the level of B7.2/MPR down regulation varied from 0 to 25% (mean 8%). This suggests that that presence of the MPR is not sufficient to make a membrane protein undergo efficient PMA-induced shedding.

Indirect evidence for basal cleavage of the B7.2/MPR chimera, however, is shown in Figure 6b. B7.2 and B7.2/MPR transfectants were incubated in fresh medium in the absence or presence of the protease inhibitor. Two hours later, the cells were stained with anti-B7.2 antibody for FACS analysis. The expression of the wildtype B7.2 molecule did not change over time. In contrast, B7.2 fluorescence increased in the cells expressing the B7.2/MPR chimera (1.7-fold increase in mean fluorescence intensity). This suggests that the B7.2 molecule expressing the membrane-proximal sequence of L-selectin undergoes basal shedding, and that blocking the protease thereby increases its level of expression on the cell surface. Similar treatment of the wt L-selectin transfectant with the protease inhibitor resulted in an increase in fluorescence intensity of 40% (data not shown). This smaller increase is probably due to a slower rate of synthesis of this molecule.

### DISCUSSION

There is an ever lengthening list of cell surface proteins whose expression is regulated by endoproteolytic cleavage (Hooper *et al.*, 1997). This release sometimes generates a soluble molecule with important biological activity, as is the case for the shedding of membrane-associated TNF-α and proTGF-α (Peschon *et al.*, 1998). Indeed, the soluble ectodomain of L-selectin retains ligand-binding activity (Dailey, 1993), and it has been proposed that this might regulate the interaction of lymphocytes with endothelium (Schleiffenbaum *et al.*, 1992). The shedding of L-selectin is particularly remarkable because of its rapidity and its near completeness. Analysis of the specificity of human L-selectin cleavage using transfected cell lines demonstrated a cleavage site eleven amino acids external to the cell membrane and showed further that extensive amino acid substitution within the surrounding amino acids has little effect on shedding (Kahn *et al.*, 1994; Migaki *et al.*, 1995). In the current report, we determined the cleavage site of murine L-selectin and did so using normal lymphocytes, not cell lines. The results showed that cleavage occurs between R321 and S322, a site that is homologous to that of the human molecule. Again like human L-selectin, our analysis of mutations adjacent to the cleavage site demonstrated limited sequence specificity.

Our previous demonstration of shed antigen in the supernatant of normal lymphocytes showed that the normal turnover of L-selectin occurs through slow constitutive shedding (Jung and Dailey, 1990). Thus, addition of PMA or treatment of T cells with anti-CD3 results in a dramatically increased rate of cleavage (Chao *et al.*, 1997; Jung and Dailey, 1990). Interestingly, the point mutation phenylalanine to proline in the MPR (F323P), which would induce a marked change in tertiary structure, does not inhibit constitutive cleavage, but it completely blocks the ability of the cells to up regulate cleavage in response to PMA. Thus, basal and activated shedding are separable processes. While the basal shedding represents the mechanism of normal selectin turnover, rapid shedding may be the correlate of events occurring during the interaction of lymphocytes with HEV endothelial cells, perhaps facilitating transition to firm adhesion or allowing for de-adhesion after undergoing transendothelial migration.

Since most cell surface proteins are not shed after PKC activation, the proteolytic machinery must have considerable substrate specificity, even if that specificity is not defined by the primary structure at and around the cleavage site. This raises the question of how this recognition takes place. The results suggesting that the B7.2/MPR chimeric molecule undergoes basal shedding and very inefficient PMA-induced shedding implies that the MPR by itself is sufficient to render a protein susceptible to metalloproteolytic cleavage in the cell membrane. This is consistent with the finding that a chimeric construct with the MPR, transmembrane region and cytoplasmic tail of L-selectin attached to alkaline phosphatase can be cleaved by the enzyme on cell membranes (Feehan *et al.*, 1996).

Basal proteolytic cleavage, which proceeds normally in the F323P proline substitution mutant, has less stringent recognition requirements than the PMA-stimulated rapid cleavage. The poor PMA inducibility of cleavage of the B7.2/MPR chimera suggests that a region distant from the substrate cleavage site must be recognized during the activated shedding process, probably by the protease itself. In intact L-selectin, one possibility for this remote recognition site is the EGF domain, which could be a docking site for the protease, as has been suggested previously (Dailey, 1993). EGF-like sequences expressed elsewhere regulate protein-protein interactions and the activity of other proteases (Campbell and Bork, 1993). The interaction of the protease with the MPR of L-selectin is weaker than that with the substrate site of TNFα (Peschon *et al.*, 1998). It is therefore possible that, upon activation of proteolysis, a portion of the enzyme recognizes the EGF domain, increasing the overall affinity of the interaction and increasing the rate of cleavage at the separate active site. The nature of the process responsible for activating proteolysis is completely unknown, and could involve changes (e.g., phosphorylation) of the protease itself, of a protease-associated molecule, or a topological change in L-selectin that exposes the putative recognition site in the EGF domain. The inability of the F323P mutant to down regulate in response to PMA could reflect the inability of this molecule to undergo the conformational change required for increased susceptibility to proteolytic attack.

Proteolytic shedding results in a rapid loss of cell surface L-selectin, which must have a dramatic influence on leukocyte migration. It remains to be determined how L-selectin and other plasma membrane substrates are recognized by this enzyme, how the process is regulated, and the physiologic role of this cleavage in L-selectin-mediated cell traffic in vivo.

***Acknowledgements***

This work was supported by grant R01-AI22730 from the NIH.

### *References*

Arbones, M.L., Ord, D.C., Ley, K., Ratech, H., Maynard-Curry, C., Otten, G., Capon, D.J. and Tedder, T.F. (1994) Lymphocyte homing and leukocyte rolling and migration are impaired in L-selectin-deficient mice. Immunity, **1**: 247–260.

Black, R.A., Rauch, C.T., Kozlosky, C.J., Peschon, J.J., Slack, J.L., Wolfson, M.F., Castner, B.J., Stocking, K.L., Reddy, P., Srinivasan, S., Nelson, N., Boiani, N., Schooley, K.A., Gerhart, M., Davis, R., Fitzner, J.N., Johnson, R.S., Paxton, R.J., March, C.J. and Cerretti, D.P. (1997) A metalloproteinase disintegrin that releases tumour-necrosis factor-alpha from cells. Nature, **385**: 729–733.

Buxbaum, J.D., Liu, K.N., Luo, Y., Slack, J.L., Stocking, K.L., Peschon, J.J., Johnson, R.S., Castner, B.J., Cerretti, D.P. and Black, R.A. (1998) Evidence that tumor necrosis factor alpha converting enzyme is involved in regulated alpha-secretase cleavage of the Alzheimer amyloid protein precursor. J. Biol. Chem., **273**: 27765–27767.

Campbell, I.D. and Bork, P. (1993) Epidermal growth factor-like modules. Curr. Opin. Strut. Biol., **3**: 385–392.

Catalina, M.D., Carroll, M.C., Arizpe, H., Takashima, A., Estess, P. and Siegelman, M.H. (1996) The route of antigen entry determines the requirement for L-selectin during immune responses. J. Exp. Med., **184**: 2341–2351.

Chao, C.C., Jensen, R. and Dailey, M.O. (1997) Mechanisms of L-selectin regulation by activated T cells. J. Immunol., **159**: 1686–1694.

Chen, A., Engel, P. and Tedder, T.F. (1995) Structural requirements regulate endoproteolytic release of the L-selectin (CD62L) adhesion receptor from the cell surface of leukocytes. J. Biol. Chem., **182**: 519–530.

Dailey, M.O. (1993) The selectin family of cell adhesion molecules. In Lymphocyte Adhesion Molecules. Shimizu, Y., Ed. (R.G. Landes Co.), pp. 75–104.

Feehan, C., Darlak, K., Kahn, J., Walcheck, B., Spatola, A.F. and Kishimoto, T.K. (1996) Shedding of the lymphocyte L-selectin adhesion molecule is inhibited by a hydroxamic acid-based protease inhibitor. Identification with an L-selectin-alkaline phosphatase reporter. J. Biol. Chem., **271**: 7019–7024.

Hafezi-Moghadam, A. and Ley, K. (1999) Relevance of L-selectin shedding for leukocyte rolling in vivo. J. Exp. Med., **189**: 939–948.

Hooper, N.M., Karran, E.H. and Turner, A.J. (1997) Membrane protein secretases. Biochem. J., **321**: 265–279.

Huang, K., Beigi, M. and Daynes, R.A. (1990) Peripheral lymph node-specific and Peyer's patch-specific homing receptors are differentially regulated following lymphocyte activation. Reg. Immunol., **3**: 103–111.

Jung, T.M. and Dailey, M.O. (1990) Rapid modulation of homing receptors ($gp90^{MEL-14}$) induced by activators of protein kinase C. Receptor shedding due to accelerated proteolytic cleavage at the cell surface. J. Immunol., **144**: 3130–3136.

Jutila, M.A., Kishimoto, T.K. and Butcher, E.C. (1990) Regulation and lectin activity of the human neutrophil peripheral lymph node homing receptor. Blood, **76**: 178–183.

Kahn, J., Ingraham, R.H., Shirley, F., Migaki, G.I. and Kishimoto, T.K. (1994) Membrane proximal cleavage of L-selectin: identification of the cleavage site and a 6-kD transmembrane peptide fragment of L-selectin. J. Cell Biol., **125**: 461–470.

Kahn, J., Walcheck, B., Migaki, G.I., Jutila, M.A. and Kishimoto, T.K. (1998) Calmodulin regulates L-selectin adhesion molecule expression and function through a protease-dependent mechanism. Cell, **92**: 809–818.

Kishimoto, T.K., Jutila, M.A., Berg, E.L. and Butcher, E.C. (1989) Neutrophil Mac-1 and MEL-14 adhesion proteins inversely regulated by chemotactic factors. Science, **245**: 1238–1241.

Migaki, G.I., Kahn, J. and Kishimoto, T.K. (1995) Mutational analysis of the membrane-proximal cleavage site of L-selectin: relaxed sequence specificity surrounding the cleavage site. J. Exp. Med., **182**: 549–557.

Mobley, J.L. and Dailey, M.O. (1992) Regulation of adhesion molecule expression by CD8 T cells in vivo. I. Differential regulation of gp90MEL-14 (LECAM-1), Pgp-1, LFA-1, and VLA-4 alpha during the differentiation of cytotoxic T lymphocytes induced by allografts. J. Immunol., **148**: 2348–2356.

Moss, M.L., Jin, S.L., Milla, M.E., Bickett, D.M., Burkhart, W., Carter, H.L., Chen, W.J., Clay, W.C., Didsbury, J.R., Hassler, D., Hoffman, C.R., Kost, T.A., Lambert, M.H., Leesnitzer, M.A., McCauley, P., McGeehan, G., Mitchell, J., Moyer, M., Pahel, G., Rocque, W., Overton, L.K., Schoenen, F., Seaton, T., Su, J.L. and Becherer, J.D. (1997) Cloning of a disintegrin metalloproteinase that processes precursor tumour-necrosis factor-alpha. Nature, **385**: 733–736.

Peschon, J.J., Slack, J.L., Reddy, P., Stocking, K.L., Sunnarborg, S.W., Lee, D.C., Russell, W.E., Castner, B.J., Johnson, R.S., Fitzner, J.N., Boyce, R.W., Nelson, N., Kozlosky, C.J., Wolfson, M.F., Rauch, C.T., Cerretti, D.P., Paxton, R.J., March, C.J. and Black, R.A. (1998) An essential role for ectodomain shedding in mammalian development. Science, **282**: 1281–1284.

Pircher, H., Mak, T.W., Lang, R., Ballhausen, w., Ruedi, E., Hengartner, H., Zinkernagel, R.M. and Burki, K. (1989) T cell tolerance to $Mls^a$ encoded antigens in T cell receptor Vβ8.1 chain transgenic mice. EMBO J., **8**: 719–727.

Preece, G., Murphy, G. and Ager, A. (1996) Metalloprotease-mediated regulation of L-selectin levels on leukocytes. J. Biol. Chem., **271**: 11634–11640.

Saiki, R.K., Gelfand, D.H., Stoffel, S., Scharf, S.J., Higuchi, R., Horn, G.T., Mullis, K.B. and Erlich, H.A. (1988) Primer-directed enzymatic amplification of DNA with a thermostable DNA polymerase. Science, **239**: 487–491.

Schleiffenbaum, B., Spertini, O. and Tedder, T.F. (1992) Soluble L-selectin is present in human plasma at high levels and retains functional activity. J. Cell Biol., **119**: 229–238.

Steeber, D.A., Green, N.E., Sato, S. and Tedder, T.F. (1996) Humoral immune response in L-selectin-deficient mice. J. Immunol., **157**: 4899–4970.

Tedder, T.F., Steeber, D.A. and Pizcueta, P. (1995) L-selectin-deficient mice have impaired leukocyte recruitment into inflammatory sites. J. Exp. Med., **181**: 2259–2264.

Walcheck, B., Kahn, J., Fisher, J.M., Wang, B.B., Fisk, R.S., Payan, D.G., Feehan, C., Betageri, R., Darlak, K., Spatola, A.F. and Kishimoto, T.K. (1996) Neutrophil rolling altered by inhibition of L-selectin shedding in vitro. Nature, **380**: 720–723.

Xu, J., Grewal, I.S., Geba, G.P. and Flavell, R.A. (1996) Impaired primary T cell responses in L-selectin-deficient mice. J. Exp. Med., **183**: 589–598.

